# Primary leiomyosarcoma of the seminal vesicle: Case report and review of the literature

**DOI:** 10.1186/1471-2407-11-323

**Published:** 2011-07-29

**Authors:** Cécile Cauvin, Laurence Moureau-Zabotto, Bruno Chetaille, Werner Hilgers, Yves Denoux, Jocelyne Jacquemier, Jérôme Guiramand, Anthony Sarran, François Bertucci

**Affiliations:** 1 Department of Radiotherapy, Institut Paoli-Calmettes, 27 bd Leï Roure, 13009, Marseille, France; 2 Department of Pathology, Institut Paoli-Calmettes, 27 bd Leï Roure, 13009, Marseille, France; 3 Department of Medical Oncology, Institut Sainte-Catherine, 1750 ch du Lavarin, 84082, Avignon, France; 4 Department of Pathology, Hôpital Foch, 40 rue Worth, 92151 Suresnes, France; 5 Department of Surgical Oncology, Institut Paoli-Calmettes, 27 bd Leï Roure, 13009, Marseille, France; 6 Department of Radiology, Institut Paoli-Calmettes, 27 bd Leï Roure, 13009, Marseille, France; 7 Department of Medical Oncology, Institut Paoli-Calmettes, 27 bd Leï Roure, 13009, Marseille, France; 8 University of Mediterranea, 58 bd Charles Livon, 13001, Marseille, France

**Keywords:** leiomyosarcoma, seminal vesicle

## Abstract

**Background:**

Primary leiomyosarcoma of the seminal vesicle is exceedingly rare.

**Case Presentation:**

We report a case of a 59-year-old man with tumour detected by rectal symptoms and ultrasonography. Computed tomography and magnetic resonance imaging suggested an origin in the right seminal vesicle. Transperineal biopsy of the tumour revealed leiomyosarcoma. A radical vesiculo-prostactectomy with bilateral pelvic lymphadenectomy was performed. Pathological examination showed a grade 2 leiomyosarcoma of the seminal vesicle. The patient received adjuvant radiotherapy. He developed distant metastases 29 months after diagnosis, and received chemotherapy. Metastatic disease was controlled by second-line gemcitabine-docetaxel combination. Fifty-one months after diagnosis of the primary tumour, and 22 months after the first metastases, the patient is alive with excellent performance status, and multiple asymptomatic stable lung and liver lesions.

**Conclusions:**

We report the eighth case of primary leiomyosarcoma of the seminal vesicle and the first one with a so long follow-up.

## Background

Less than 5% of soft tissue sarcomas (STS) arise from the genitourinary tract. Leiomyosarcomas of the seminal vesicle are exceedingly rare. To our knowledge, only seven cases have been reported to date in the English literature and all with a short follow-up [1-6]. Diagnosis, initially described as difficult because of invaded surrounding structures, which obscure the actual place of origin, is being facilitated by modern imaging techniques such as computed tomography and magnetic resonance imaging. Data on optimal treatment are limited, but radical surgery seems essential. The role of adjuvant chemotherapy and radiotherapy remains unclear. Here, we report an additional case of primary leiomyosarcoma of the seminal vesicle with a long follow-up of more than 4 years, and review the literature.

## Case presentation

The patient was a 59-year old man, Caucasian type, without any specific medical personal or familial history. In October 2006, he underwent a pelvic ultrasonography (US) because he presented a "pressure sensation" in the rectum and rectal imperiosity for four weeks. Physical examination revealed no additional symptom, except the rectal examination that detected a 4-cm hard pre-rectal mass filling the right side of the pelvis. The WHO performance status was equal to 0. Ultrasonography (US) discovered a tumour located at the right side of the pelvis. Serum prostate-specific antigen levels were normal. The patient was thus referred to hospital.

**Figure 1:**
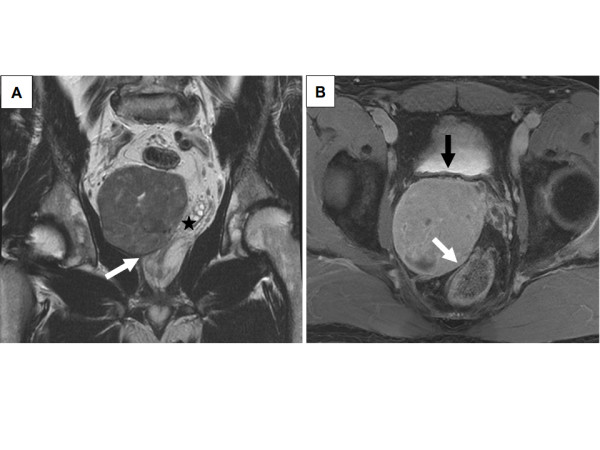
**Magnetic resonance imaging of the pelvis**. A/Coronal T2-weighted MRI showing an heterogeneous mass lesion in the region of right seminal vesicle, causing inferiorly and to the left displacement of the prostate (white arrow) and of the left seminal vesicle which appears normal (star). B/Axial contrast-enhanced T1-weighted MRI showing distinct margins between the mass and both the posterior wall of the bladder (black arrow) and the anterior wall of the rectum (white arrow).

Computed tomography (CT) of abdomen and pelvis revealed an 8-cm heterogeneous tumour predominantly located on the right side of the pelvis likely arising from the right seminal vesicle, reaching the median line and adherenting to the posterior bladder wall and the anterior rectal wall. Due to the rectal clinical symptoms and the suspicion of rectal adherence on CT scan, the core needle biopsy was CT-guided transperineal rather than transrectal. Pathological analysis revealed a well-differentiated leiomyosarcoma. Pelvic magnetic resonance imaging (MRI) confirmed the presence of a mass centred on the right seminal vesicle, causing mass effect on the prostate and left seminal vesicle, without a cleavage plane with the right prostate and the right obturator muscle, suggesting invasion (Figure 1). Otherwise, MRI clearly showed fat interface between the mass, the posterior bladder wall and the anterior rectal wall without any sign of involvement. Complete clinical and radiological screening did not detect any lymphadenopathy or distant metastasis. Based on these data, the tumor was considered completely resectable, and surgery was decided.

**Figure 2:**
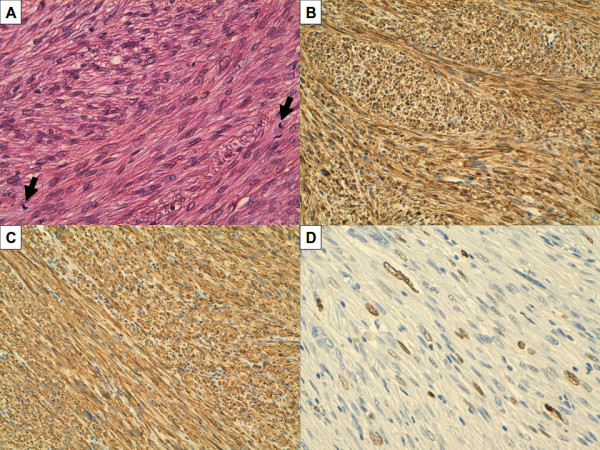
**Well-differentiated leiomyosarcoma**. A/H&E staining showing intersecting fascicles of moderately atypical elongated cells with abundant eosinophilic cytoplasm. Mitoses (black arrows) are seen (original magnification X 200). B-D/Immunohistochemical analysis showing that tumour cells strongly express smooth-muscle actin (B, X 200) and H-caldesmone (C, X 200). 15% of the cells are stained by Ki-67 proliferative marker (D, X 200).

Before surgery, the examination under general anesthesia revealed that the mass was difficult to mobilize. The treatment consisted of a radical vesiculo-prostatectomy with limited (ilio-obturator and hypogastric) bilateral pelvic lymphadenectomy in January 2007, associated with resection of the internal obturator muscle. No anterior rectal resection was necessary. Excision was macroscopically complete. The post-operative course was uneventful. The pathological macroscopic examination of the surgical resection specimen showed a small prostate (4.5 × 4 × 3 cm) and an 8 × 8 × 6.5 cm well-limited hard white-tan mass, centred on the right seminal vesicle, tangent to the prostate base, pushing the vas deferens, the prostate and the left seminal vesicle. On cut section the mass showed a grey-white whorled appearance with foci of necrosis. Microscopic analysis (Figure 2A) confirmed the previous diagnosis by showing intersecting fascicles of atypical spindle cells with elongated blunt-ended nuclei and eosinophilic cytoplasm very reminiscent of smooth muscle differentiation. Immunohistochemical analysis showed strong positive staining of the tumour cells for smooth-muscle actin and H-caldesmone (Figure 2B-D), and negative staining for pan-cytokeratin AE1/AE3, CD117. The mitotic rate averaged ten mitoses per ten high-power fields. Necrosis was present on less than 50% of the tumour surface. The tumour seemed developed within the wall of the right seminal vesicle. It was a well-limited and pseudo-wrapped with limited surgical margins, sometimes inferior to 1 mm. The retained diagnosis was moderately differentiated (FNCLCC grade 2) leiomyosarcoma of the right seminal vesicle. The fourteen removed pelvic lymph nodes were free of tumour. Moreover, an associated small (4 × 4 × 2 mm) prostatic adenocarcinoma was fortuitously discovered on the left apex of the gland, Gleason 6 (3+3), without any macro- and microscopical connection with the sarcoma. After surgery and because of closed margins for the leiomyosarcoma, the patient received adjuvant pelvic external beam radiation therapy achieved in April 2007 (56 Gy./31 fractions). No adjuvant chemotherapy was delivered. The patient was then regularly monitored at the clinical and radiological levels.

**Figure 3:**
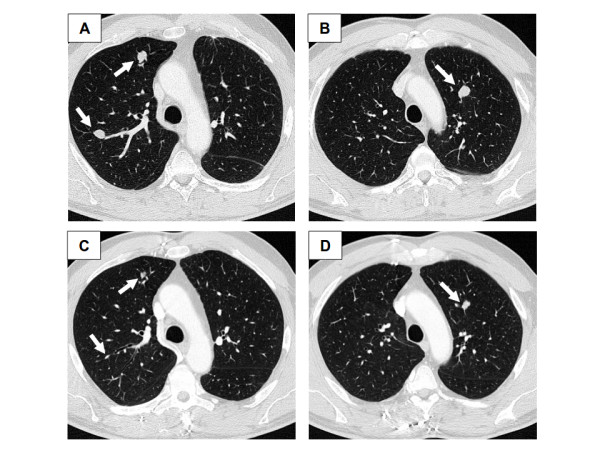
**Response of lung metastases to gemcitabine-docetaxel chemotherapy**. Chest CT-scan performed in October 2009 (A-B) and in August 2010 (C-D) showing partial response of lung metastases (arrows) after 9 cycles of second-line gemcitabine-docetaxel combination.

In April 2009, 29 months after diagnosis, he developed two subcutaneous nodules on the scalp. Surgical removal and pathological analysis confirmed the diagnosis of distant recurrences of leiomyosarcoma. At the same time, CT and TEP scans detected the presence of multiple asymptomatic liver and lung metastases. Performance status was excellent (WHO 0). First-line chemotherapy consisted of six cycles combining doxorubicin and ifosfamide, but metastases progressed (October 2009), and the patient was referred to our institution for second-line chemotherapy. We delivered a gemcitabine-docetaxel combination. After three cycles, CT scan showed stable lung and liver lesions, whose size decreased after the sixth cycle, then after the ninth cycle, with a 30% response when compared with October 2009 (Figure 3). Treatment was then interrupted, and the patient regularly followed. A last visit, in February 2011, 51 months after diagnosis of the primary tumour, and 22 months after the first metastasis, the patient is alive with excellent performance status, without any symptom, and with multiple stable lung and liver lesions.

## Discussion

Primary malignant tumours of the seminal vesicles are very rare. Among them, carcinomas are much more frequent than sarcomas [7]. Primary leiomyosarcoma of the seminal vesicle is extremely rare with to our knowledge only seven cases reported in the English literature. Our case represents the eighth patient, and the first one with a follow-up superior to 30 months.

All cases are summarized in Table 1. The median age of patients at the time of diagnosis is 59,5 years (range, 37 to 68). No specific risk factor has been identified. Clinical detection of seminal vesicle tumours is difficult. Depending on the size and extension of the tumour, patients are either asymptomatic with discovery of the lesion by palpation on routine rectal examination (3 cases), or present non-specific urinary and/or rectal symptoms (5 cases). Of note, no case with hemospermia, hematuria or anejaculation has been reported.

**Table 1:** Eight cases of leiomyosarcoma of the seminal vesicle reported in literature

Reference	Age (years)	Clinical symptoms	Diagnostic biopsy	Initial treatment	Pathological margins	Pathological tumor size	Tumor grade	Adjuvant treatment	Clinical outcome
[5]	60	none (RE)	yes	surgery	R0	3,5 cm	intermediate	no	14 months: no relapse, alive
[4]	NA	none (RE)	NA	surgery	R1	NA	high	no	24 months: no relapse, alive
[4]	NA	none (RE)	NA	surgery	R1	NA	high	no	29 months: metastasis (lung), alive with disease (doxorubicin)
[2]	68	rectal and pain	yes	surgery	R0	10 cm	high	no	13 months: no relapse, alive
[3]	64	urinary	no	surgery	R0	8 cm	high	no	24 months: metastasis (kidney), alive with disease (CT)
[6]	46	urinary and pain	yes	surgery	NA	NA	NA	no	6 months: no relapse, alive
[1]	37	urinary and rectal	yes	surgery	NA	15 cm	intermediate	CT (MAID) and RT	20 months: no relapse, alive
Our case	59	rectal	yes	surgery	R1	8 cm	intermediate	RT	29 months: metastases (sub-cutaneous, lung, liver: MAI); 51 months: alive with disease (gemcitabine-docetaxel)

RE, rectal examination; NA, not available; CT, chemotherapy; MAID, mesna + doxorubicin + ifosfamide + dacarbazine; RT, radiation therapy.

Radiological imaging is useful for the diagnosis and staging of seminal vesicle tumours, notably to locate the initial site of tumour development. Indeed, these tumours tend to invade neighbouring structures in the retrovesical space, causing difficulty in determining the organ of origin, and leading to radical resection of several major organs. Generally the first exam is US either transrectal or transabdominal. More frequently, pelvic CT and above all MRI allow the localisation of the tumour in the seminal vesicle and a better appreciation of the locoregional extension before surgery. In our case, and as previously reported [6], MRI findings strongly suggested a tumour origin in the right seminal vesicle, followed by extension to adjacent organs (prostate, internal obturator muscle).

Sarcoma diagnosis is provided by pathological analysis of the tumour sample, either after needle biopsy (5 cases including ours), or after surgical resection (1 case). In all cases, definitive diagnosis was done by histological examination of the resected tumour. In our case, the finding of a small adenocarcinoma raised the possibility of a sarcomatoid carcinoma but the macroscopical presentation of a clearly extraprostatic tumour, the absence of any connection between the two proliferations, and negativity of epithelial markers by immunochemistry did not favour this diagnosis. Differential diagnosis also concerns leimoyosarcoma arising from adjacent organs (prostate, bladder, rectum) and secondarily invading the seminal vesicle. But today, thanks to the advent of modern imaging tools, the organ of origin can be documented in most of cases by looking at the epicentre of the mass and the pattern of displacement of adjacent organs [6].

The prognosis of leiomyosarcoma of the seminal vesicle is poor, and more unfavourable than other urological sarcoma arising from the bladder or paratesticular site [4]. This is partly due to the uncommon form of presentation, delay in diagnosis, and difficulty in complete surgical excision. This overall poor prognosis is demonstrated in Table 1. Indeed, the median follow-up of the 8 cases is short (22 months; range, 6 to 51), and of note, the 3 patients with a follow-up superior to 24 months all experienced metastatic relapse at 24, 29, and 29 months. The 5 other patients, alive without any relapse, present a median follow-up of only 14 months (range, 6 to 24). Our case represents the one with the longest follow-up (51 months). Obviously, the small number of cases precludes any prognostic analysis. Metastatic relapses occurred in the 3 patients with microscopically incomplete resection (2 cases) and/or high grade (2 cases) and/or tumour size superior to 5 cm (2 cases), suggesting that those sarcomas share prognostic features with STS arising from other sites.

Data on optimal treatment are obviously limited. The main treatment is surgery, which consisted of a cystoprostatectomy with a pelvic lymphadenectomy in all reported cases, except ours where vesiculo-prostatectomy was done without cystectomy and without any local relapse during the follow-up. As usually in STS, only complete resection of the tumour offers a chance for a cure. In the literature, the margins were positive in only 2 cases. In our case the margins were very close, less than 1 mm, justifying the use of adjuvant radiation therapy. The role of adjuvant radiation therapy in visceral STS is not established [8], and only two out of 8 reported cases, including ours with very close margins, received such treatment. Of note, none of the 8 patients, including ours treated without cystectomy, experienced any local relapse during the available follow-up. Regarding adjuvant chemotherapy, its role in STS remains unclear [9], and only one patient received adjuvant combination of doxorubicin, ifosfamide and dacarbazine (MAID). Anthracycline-based chemotherapy was offered to the 3 patients at the time of metastatic relapse, but the response is documented in our case only. Our patient's disease progressed after 6 doxorubicin-ifosfamide cycles, but responded to second-line chemotherapy based on gemcitabine and docetaxel, as observed with leiomyosarcomas arising from other sites [10].

## Conclusions

We report the eighth case of primary leiomyosarcoma of the seminal vesicle with the longest follow-up. As these tumours are exceptional and information about standard treatment is lacking, we think that case reports such as this one may serve as the only reference for clinicians taking care of these patients. Radical surgical excision is the best chance for cure, but the need for cystectomy must be discussed. Furthermore, like leiomyosarcomas from other sites, a multimodality treatment deserves to be discussed and systemic therapy to be improved given the metastatic risk and overall poor prognosis. In this context, the gemcitabine-docetaxel combination is likely so efficient than in other locations.

## Consent

Written informed consent was obtained from the patient (February 2011) for publication of this case report and any accompanying images. A copy of the written consent is available for review by the Editor-in-Chief of this journal.

## Abbreviations

STS: soft tissue sarcoma; US: ultrasonography; WHO: World Health Organisation; CT: computed tomography; MRI: magnetic resonance imaging; FNCLCC: Fédération Nationale des Centres de Lutte Contre le Cancer.

## Competing interests

The authors declare that they have no competing interests.

## Authors' contributions

Conception and design: CC, LMZ and FB. Manuscript writing: CC, LMZ and FB. Final approval: FB, CC, LMZ, BC, WH, YD, JJ, JG, AS. Pathological explorations: BC, YD, JJ. Patient's management: FB, LMZ, WH, AS, JG.

## Pre-publication history

The pre-publication history for this paper can be accessed here:

http://www.biomedcentral.com/1471-2407/11/323/prepub
